# Expanding access to safe ambulatory manual vacuum aspiration abortion up to 14+6 weeks following Argentina's legal reform: an observational study in the public health sector

**DOI:** 10.1186/s12978-025-02036-8

**Published:** 2025-05-24

**Authors:** Biani Saavedra-Avendano, María Paula Botta, Dolores Chaumet, Berenice Macagno, Guillermo Antonio Ortiz-Avendano

**Affiliations:** 1 Ipas LAC, Mexico City, Mexico; 2 CEMAR, Rosario, Santa Fe Argentina; 3 Ipas US, Chapel Hill, North Caroline USA

**Keywords:** Manual vacuum aspiration, Primary care, Local anesthesia, Ambulatory care, Legal change

## Abstract

**Background:**

In December 2020, Argentina passed Law 27.610, legalizing elective abortion up to 14 + 6 weeks and beyond in cases of rape or health risks. This study aims to analyze the sociodemographic characteristics of users who opted or were referred for manual vacuum aspiration (MVA) services at an a ambulatory medical center in Argentina before and after the legal reform, and to assess the safety and effectiveness of outpatient MVA procedures for pregnancies up to 14 + 6 weeks.

**Methods:**

Observational study using clinical data from patients up to 14 + 6 weeks gestation (*n* = 1,861) who sought or were referred for outpatient MVA abortion at a public healthcare facility in Rosario, Argentina (2017–2023). We analyze changes in users’ sociodemographic characteristics before and after the legal reform and assess the safety and effectiveness of ambulatory MVA abortion by gestational age (< 13 weeks vs. 13–14 weeks). A logistic regression tested associations between sociodemographic, procedural, and reproductive factors, and receiving MVA after 12 weeks.

**Results:**

Of the 1,861 MVA abortions, 85% (*n* = 1,590) were provided before 13 weeks’ gestation, and 15% (*n* = 271) occurred between 13–14 weeks. After the legal reform, more users accessed outpatient MVA services beyond 12 weeks (7% vs. 22%; *p* < 0.05: before and after the legal change, respectively), including individuals with lower education levels (46% vs. 54%; *p* < 0.05:), informal employment (34% vs. 47%; *p* < 0.05), without healthcare insurance (72% vs. 90%; *p* < 0.05), and nulliparity (18% vs. 30%; *p* < 0.05). The success rate of ambulatory MVA abortion was 99.9%, with 0.4% (*n* = 7) adverse events; no statistically significant differences by gestational age groups (< 13 weeks vs. 13–14 weeks). The legal reform was positively associated with accessing MVA abortion after 12 weeks.

**Conclusions:**

The legal reform improved access to safe ambulatory MVA abortion services up to 14 + 6 weeks’ gestation, particularly for socially disadvantaged users. MVA abortion, both before 13 weeks and at 13–14 weeks, demonstrated a high success rate (99.9%) with minimal adverse events.

## Plain English summary

In December 2020, Argentina approved Law 27.610, allowing voluntary induced abortion up to 14 + 6 weeks of pregnancy and later in cases of rape or health risks. This study looks at the characteristics of people who chose or were referred to manual vacuum aspiration (MVA) services at an outpatient medical center in Argentina before and after the law changed. It also assesses how safe and effective outpatient MVA procedures are for pregnancies up to 14 + 6 weeks. We reviewed the clinical records of 1,861 patients up to 14 + 6 weeks of gestation who requested or were referred for MVA abortion at a public healthcare facility in Rosario, Argentina, between 2017 and 2023. We analyzed changes in users' sociodemographic characteristics before and after elective abortion decriminalization and assessed the safety and effectiveness of outpatient MVA abortions for pregnancies under 13 weeks compared to 13–14 weeks. Our results show that after elective abortion decriminalizing up to 14 + 6 weeks in Argentina, access to safe ambulatory MVA services increased, benefiting the most socially vulnerable populations. It also demonstrates the safety and effectiveness of MVA in first-level ambulatory services for pregnancies up to 14 + 6 weeks compared to those under 13 weeks.

## Introduction

Over the past two decades, regulatory changes related to abortion have occurred across numerous Latin American countries, resulting in expanded rights but occasionally imposing restrictions [1, 2]. Legal frameworks are a critical factor in shaping abortion access [3, 4]. Abortion decriminalization improves maternal health by reducing unsafe procedures and also contributes to lowering fertility, particularly among adolescents [5, 6]. However, a common issue in many countries is the noticeable disparity between the development of laws and their practical implementation [1]. Access to abortion depends on technical guidelines, service availability, trained personnel, and supplies. Some countries in the region have regulations that act as access barriers, such as requiring specific clinical spaces, specialized personnel, mandatory diagnostic studies, waiting periods, medical committees, third-party consent, or judicial authorization [2]. These demands for specialized facilities and personnel constitute a barrier to access and contradict evidence-based recommendations [7].

In December of 2020, Argentina passed Law 27.610, which came into effect in January 2021 [8, 9]. This legislation allows for Voluntary Interruption of Pregnancy (*Interrupción Voluntaria del Embarazo* IVE, in Spanish) based on the individual's decision up to 14 + 6 weeks gestation and Legal Interruption of Pregnancy (*Interrupción Legal del Embarazo* ILE, in Spanish) from the 15 th week onward in cases of rape, threats to life, or health [8, 9]. Prior to the decriminalization of voluntary abortion, access was granted through legal exceptions [10]. Law 27.610 represents a significant legal advance for Latin America and the Caribbean region. In comparison, territories that had previously decriminalized abortion at the woman's or pregnant individual's request, such as Mexico City and several other Mexican states, as well as Uruguay, established a gestational limit of 12 weeks. Argentina extended the voluntary abortion limit to 14 + 6 weeks.

In general, abortions after 12 weeks of gestation represent a small percentage of all abortions worldwide (between 10 and 15%). However, studies have identified characteristics of women and other pregnant individuals associated with seeking voluntary abortion care after the first trimester. For example, being an adolescent [11, 12], facing adverse socioeconomic conditions such as lack of health insurance [13], having low educational attainment and income [14, 15], as well as logistical challenges in accessing services [16] and social stigma surrounding abortion [17].

The technical guide for voluntary legal abortions, issued by the Ministry of Health of Argentina [18] in accordance with the World Health Organization's abortion care guidelines [7], recommends the manual vacuum aspiration (MVA) technique as a safe abortion procedure up to 14 + 6 weeks of gestation, providing an alternative to medication-based treatments [19]. MVA is a low-cost and portable procedure, making it more accessible than electric vacuum aspiration, especially in primary care settings or low-resource settings [19]. The safety and efficacy of MVA for first-trimester abortion care are well established [20]. However, there is limited literature documenting the safety and efficacy of MVA beyond 12 weeks in outpatient healthcare services in the Latin American context.

The objective of this study is to analyze the sociodemographic characteristics of elective MVA patients before and after the abortion legal reform, and to assess the safety and effectiveness of ambulatory MVA procedures for pregnancies up to 14 + 6 weeks’ gestation in a public primary healthcare facility.

## Methods

We conducted an observational study using data from the Safe Abortion Access Network (Red de Acceso al Aborto Seguro, REDAAS in Spanish) database [21]. REDAAS is a network of health professionals working in Argentina's public health services. To gather data on abortion, its members develop databases containing information about the patients they serve. We included information of *n* = 1,861 outpatient MVA abortions provided at the Center for Ambulatory Medical Specialties in Rosario (Centro de Especialidades Médicas Ambulatorias, CEMAR in Spanish) from 2017 to 2023.

### Setting

Rosario is the largest city in the central Argentine province of Santa Fe and the third-most populous city in the country after Buenos Aires and Córdoba. Since 2016, CEMAR in Rosario has been a reference center for abortion care using manual vacuum aspiration MVA. Since then, it has been providing outpatient MVA abortion services with local anesthesia. After the changes in abortion laws in 2020, CEMAR continued to serve as a referral center, with approximately 85% of its patients referred from local health centers and municipal hospitals [22]. CEMAR provides the highest number of MVA procedures in within the public healthcare system, due to its larger human resources and reception capacity.

### ,Variables and statistical analysis

To identified changes in MVA users’ sociodemographic characteristics, our primary independent variable was changes in abortion laws. The “pre-legal change” years were 2017–2020, and the “post-legal change” years were 2021–2023. Elective abortion was decriminalized in December 2020 and came into effect in January 2021 [8, 9]. We analyzed sociodemographic characteristics such as age grouped into five categories (< 19, 19–24, 25–30, 31–35, and > = 36); educational level categorized into three groups (primary, if women completed primary education or have incomplete secondary education; secondary, if they have completed secondary education or did not finish university; and university, if they achieved a university-level education); an indicator of healthcare insurance for those with healthcare coverage; occupation categorized as unpaid domestic work, formal employment, or informal employment, the number of living children divided into five categories (none, 1–2, 3–4, > = 5, or not available information); and the gestational length at the time of the procedure. We used Chi-squared tests to assess differences in those characteristics before and after the abortion laws changes in 2020.

We analyzed the percentage distribution of gestational weeks among patients who underwent the MVA procedure at CEMAR. Then we used bivariate descriptive analyses to examine procedure and reproductive information by gestational length comparison groups (< 13 vs 13–14 weeks). We assessed the effectiveness and safety of the MVA procedure, measured by MVA abortion failure (e.g., ongoing pregnancy, incomplete abortion), and observed adverse events (mild hemorrhage and precordial pain). We do not use the term'complications'because none of the observations presented an event that falls within the World Health Organization's definition of abortion complications: excessive bleeding or hemorrhage, infections, cervical injuries, or uterine perforations [7, 23, 24]. We also analyzed type of abortion procedure grouped as MVA after medication abortion (MA) failure or elective MVA. Chi-squared tests were used to assess differences between gestational groups in abortion outcomes.

We conducted a logistic regression model presented in Odds Ratio to identify factors associated with receiving MVA services after 12 weeks gestation at CEMAR. Our primary independent variable was the decriminalization of elective abortion, with a comparison between the pre-legal change years and the post-legal change years. The following sociodemographic characteristics were included as covariates: age groups, educational level, occupation, health insurance status, number of live births (categorized as 0, 1–2, and ≥ 3), and the type of abortion procedure, categorized as MVA following medication abortion (MA) failure or MVA alone.

The current research study was reviewed and approved by the members of the Research Ethics Committee of the Department of Public Health of the Municipality of Rosario No. 090834. We conducted the data analyses using the STATA 15 software.

## Results

**Fig. 1:**
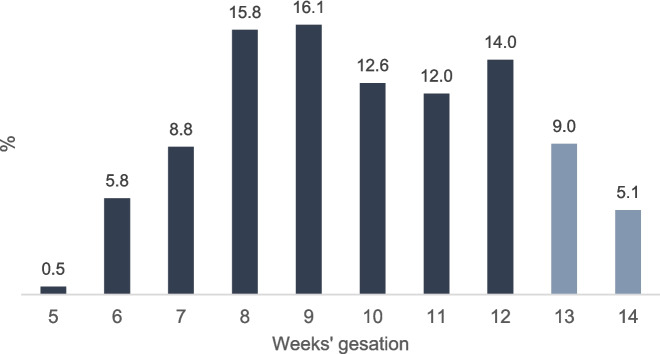
Weeks of gestation among patients receiving ambulatory MVA abortion care in the CEMAR, Argentina (2017–2023) Note: The color differences in the bars indicate the comparison groups, with deep blue representing MVA abortions ≤ 12 weeks of gestation and gray indicating procedures conducted in weeks 13 and 14.Source: Self-produced based on data from the Safe Abortion Access Network (REDAAS)

During the study period 2017–2023, our sample included 1,861 MVA abortion procedures with 49% occurring before and 51% occurring after abortion decriminalization. 86% of our sample sought care before 13 weeks’ gestation and 14% during weeks 13 and 14 of gestation (Table 1). Figure 1 depicts the distribution gestational weeks, week by week. The highest frequency was observed at 9 weeks (16%). Meanwhile, 9% of the sample sought abortion in week 13, and only 5% in week 14 of gestation.

**Table 1:** Sociodemographic characteristics of patients receiving ambulatory MVA abortion in the CEMAR, Argentina before and after the abortion decriminalization by weeks’ gestation (2017–2023)

	**Abortion law change**					
	**Pre-legal change**			**Post-legal change**		
	< = 12 (*n* = 845)	13–14 (*n* = 62)	**Total (*n* = 907)**	< = 12 (*n* = 745)	13–14 (*n* = 209)	**Total (*n* = 954)**
	45%	3%	**49%**	40%	11%	**51%**
	Col %			Col %		
Age groups**						
< 19	10	15	**10**	5	9	**6**
19–24	31	36	**32**	35	41	**37**
25–30	31	24	**30**	32	27	**31**
31–35	17	11	**17**	16	13	**15**
36–45	11	15	**11**	12	11	**11**
Educational level**						
Primary	46	50	**46**	53	57	**54**
Secondary	49	42	**48**	44	41	**43**
University	5	8	**6**	4	1	**3**
**Occupation**						
Unpaid Domestic work	48	47	**48**	45	51	**47**
Formal employment	19	13	**18**	7	4	**7**
Informal employment	33	40	**34**	48	45	**47**
Health insurance**						
No	71	77	**72**	90	91	**90**
Yes	29	23	**28**	10	9	**10**
Live births**						
zero	18	16	**18**	30	29	**30**
1 a 2	51	40	**50**	51	55	**52**
3 a 4	13	15	**13**	18	16	**17**
> = 5	0	2	**0**	1	1	**1**
missing	18	27	**19**	0	0	**0**

^**^ *p*-value < 0.01

Self-produced based on data from the Safe Abortion Access Network (REDAAS)

Regarding the sociodemographic characteristics in Table 1, we identified statistically significant differences in the profile of MVA service users at CEMAR before and after the decriminalization of abortion. The age distribution shifted for both adolescents under 19 and young adults aged 19 to 24. Before the legal reform, 10% of MVA users were adolescents, but this percentage dropped to 6% afterward. Conversely, the proportion of young adults (19 to 24) increased from 32 to 37%. We also observed that after the decriminalization of voluntary abortion, a greater number of individuals with lower education levels (primary school; 46% vs. 54%; *p *< 0.05), informal employment (34% vs. 47%; *p* < 0.05), lacking health insurance (72% vs. 90%; *p* < 0.05), and those who were nulliparous (18% vs. 30%; *p* < 0.05) received MVA services. Following the legal reform, a larger proportion of people accessed abortion services beyond 12 weeks of gestation (62/907 = 7% vs. 209/954 = 22%). When we focus on MVA users after 12 weeks of gestation before and after the legal change, we identified a significant shift in the proportion of users with low educational attainment (50% vs 57%; *p* < 0.05), engaged in unpaid domestic work (47% vs 51%), and without health insurance (77% vs 91%; *p* < 0.05).

Table 2 shows the results of the MVA abortion procedure (effectiveness and safety) by gestational weeks’ groups. We only identified two cases of incomplete abortions (0.1%), which occurred in the group with < 13 weeks. MVA abortion success rate was 99.9%. Out of the 1,861 procedures performed, we identified seven (0.4%) adverse events with no statistically significant differences between the comparison groups. 12.3% of the abortions were due to failure of the medication abortion method (see Table 2).

**Table 2:** MVA abortion outcomes of patients receiving ambulatory MVA abortion in the CEMAR, Argentina by gestational length groups (2017–2023)

	Weeks’ gestation						
	< = 12		13–14		Total		
	1,590	85%	271	14%	1,861	100%	
	n	%	n	%	n	%	*p*-value
**MVA abortion result**							0.559
Complete	1588	99.9	271	100	1859	99.9	
Incomplete	2	0.1	0	0	2	0.1	
**MVA abortion-related adverse events**							0.292
None	1585	99.7	269	99.3	1854	99.6	
Mild	5	0.3	2	0.7	7	0.4	
**Type of procedure**							0.465
MVA after MA	192	12.1	37	13.7	229	12.3	
MVA	1398	87.9	234	86.3	1632	87.7	

Self-produced based on data from the Safe Abortion Access Network (REDAAS)

Our logistic regression model shows that, after adjusting for individual and procedural characteristics, the change in the abortion law decriminalizing voluntary abortion is positively associated with receiving MVA services after 12 weeks of gestation (OR = 4.04; 95% CI = 2.92–5.59). We also found that, compared to adolescent users, women aged 25 to 35 had lower odds of receiving an MVA abortion after 12 weeks of gestation (Table 3).

**Table 3:** Legal, sociodemographic and abortion variables associated with received MVA abortion care after 12 weeks gestation in the CEMAR, Argentina (2017–2023)

	OR	95% Conf. Interval		*p*-value
**Abortion Law Change REF = before**				
After law change	**4.04**	**2.92**	**5.59**	**0.000**
**Age REF: < 19**				
19–24	0.68	0.41	1.14	0.144
25–30	**0.47**	**0.27**	**0.82**	**0.008**
31–35	**0.43**	**0.23**	**0.81**	**0.009**
36–45	0.57	0.29	1.11	0.1
**Educational level REF: Primary**				
Secondary	0.97	0.72	1.31	0.831
University	0.98	0.44	2.19	0.959
**Occupation REF: Unpaid domestic work**				
Formal employment	0.71	0.37	1.36	0.303
Informal employment	1.02	0.76	1.35	0.918
**Health insurance REF: None**				
Yes	1.00	0.62	1.60	0.984
**Live births REF: zero)**				
1 a 2	1.31	0.91	1.88	0.148
> = 3	1.37	0.87	2.14	0.171
**Type of procedure REF: MVA after MA**				
MVA	0.69	0.46	1.03	0.068

Observations = 1,861 Source: Self-produced based on data from the Safe Abortion Access Network (REDAAS)

## Discussion

Our study contributes to the growing body of evidence indicating that the decriminalization of abortion benefits socially vulnerable populations, including young individuals, those without access to health insurance, people with low levels of formal education, and those engaged in precarious or unpaid work [12, 25]. Moreover, our findings suggest that extending the gestational limit beyond 12 weeks—commonly stipulated by legislation across Latin American countries—offers significant advantages to the most marginalized sectors of the population who most commonly face barriers to accessing abortion services and therefore experience greater delays in receiving care [13–15, 26].

The experience at CEMAR, the main referral center for elective manual vacuum aspiration (MVA) abortions up to 14 + 6 weeks’ gestation in one of Argentina’s largest cities, demonstrates that performing the procedure on a public primary healthcare outpatient service, using local anesthesia and without the need for specialized equipment such as an operating room, is both safe and effective. We observed a 99.9% success rate for MVA abortions, with zero complications and a 0.4% rate of mild adverse events. These findings are consistent with other studies evaluating the effectiveness and safety of MVA during the first trimester, where < = 3.0% of cases required additional intervention, and < = 0.1% resulted in complications [16].

The use of local anesthesia as a paracervical block is effective for pain relief and facilitates MVA procedures, as it does not require pre-surgical studies, fasting, or the intervention of an anesthesiologist [27–29]. Additionally, it reduces the risks associated with general anesthesia and shortens hospital stay times [30]. CEMAR’s model expand access to abortion by providing a dedicated workspace that operates independently of surgical schedules, allowing for the use of local anesthesia instead of general anesthesia. This approach not only increases efficiency but also reduces the need for operating room resources, making abortion services more accessible to a larger number of individuals.

Access to abortion care at the primary level is crucial for ensuring timely care, especially for individuals who face barriers to accessing hospital services. Navigating hospital services can be challenging due to factors such as geographic distance, financial costs, lack of transportation, and the complexity of the healthcare system [31, 32]. Primary care, on the other hand, is more accessible and cost-effective, as it provides services in community-based settings. Additionally, primary care can offer comprehensive, coordinated care tailored to individual needs, which can lead to improved health outcomes and reduced healthcare costs. From a public policy perspective, our findings suggest that health systems should consider integrating MVA services into primary care settings. Doing so could significantly enhance timely access to safe abortion, particularly for underserved populations.

### Strengths and limitations

Despite its informative nature, this study has several limitations. First, it was not possible to include medication abortion procedures from CEMAR, as being a referral center means that 100% of the procedures performed are MVA abortions. Second, although CEMAR provides 85% of aspiration abortion services in Rosario, Argentina, the scope of this study was limited to a specific geographic region, which may affect the generalizability of our findings to other populations or settings. Nonetheless, our results are highly consistent with those reported in other locations.

### Interpretation

This study shows that after the decriminalization of elective abortion up to 14 + 6 weeks in Argentina, access to safe ambulatory MVA services increased, which may have particularly benefited populations facing greater barriers to care. It also shows the safety and effectiveness of MVA in first-level ambulatory services for pregnancies up to 14 + 6 weeks compared to those under 13 weeks. The high success rate (99.9%) of outpatient MVA abortions and the very low rate of complications (0%) and mild adverse events (0.4%) underscore the safety and effectiveness of this procedure for pregnancies up to 14 + 6 weeks. These results support the expansion of MVA as a reliable option for abortion care in public primary healthcare units, which can increase access to safe abortion services for a broader population. This is particularly important in contexts where access to instrumental abortion procedures is limited due to technical, technological, and equipment requirements, as well as human resources that are mostly costly, scarce, and difficult to access.

## Data Availability

The datasets generated and/or analyzed during the current study are not publicly available because the authors do not have authorization from the health department to share patient individual-level data but are available from the corresponding author upon reasonable request.
